# Mitochondria Lead the Way: Mitochondrial Dynamics and Function in Cellular Movements in Development and Disease

**DOI:** 10.3389/fcell.2021.781933

**Published:** 2022-02-02

**Authors:** Somya Madan, Bhavin Uttekar, Sayali Chowdhary, Richa Rikhy

**Affiliations:** Department of Biology, Indian Institute of Science Education and Research, Pune, India

**Keywords:** mitochondrial fusion, mitochondrial fission, epithelial cell morphogenesis, epithelial to mesenchymal transition, embryogenesis, cell migration, cell division, wound healing

## Abstract

The dynamics, distribution and activity of subcellular organelles are integral to regulating cell shape changes during various physiological processes such as epithelial cell formation, cell migration and morphogenesis. Mitochondria are famously known as the powerhouse of the cell and play an important role in buffering calcium, releasing reactive oxygen species and key metabolites for various activities in a eukaryotic cell. Mitochondrial dynamics and morphology changes regulate these functions and their regulation is, in turn, crucial for various morphogenetic processes. In this review, we evaluate recent literature which highlights the role of mitochondrial morphology and activity during cell shape changes in epithelial cell formation, cell division, cell migration and tissue morphogenesis during organism development and in disease. In general, we find that mitochondrial shape is regulated for their distribution or translocation to the sites of active cell shape dynamics or morphogenesis. Often, key metabolites released locally and molecules buffered by mitochondria play crucial roles in regulating signaling pathways that motivate changes in cell shape, mitochondrial shape and mitochondrial activity. We conclude that mechanistic analysis of interactions between mitochondrial morphology, activity, signaling pathways and cell shape changes across the various cell and animal-based model systems holds the key to deciphering the common principles for this interaction.

## Introduction

Mitochondria are double-membrane, dynamic, semi-autonomous organelles in eukaryotic cells involved in a large variety of functions regulating cellular physiology and signaling. Mitochondrial activity and shape regulation has been recently shown to be integral to cell shape changes. In this review, we discuss recent literature that evaluates the mechanistic interactions between mitochondrial function, morphology and dynamics in morphogenetic processes in selected paradigms of development and disease.

Mitochondria are well known for their function in ATP synthesis due to the redox cycles of electron transport chain (ETC) proteins in the inner mitochondrial membrane (Stroud and Ryan, 2013). ETC complexes assemble into super-complexes, increasing mitochondrial ATP generation efficiency (Schägger and Pfeiffer, 2000; Lapuente-Brun et al., 2013; Kasahara and Scorrano, 2014; Cogliati et al., 2016). In addition to ATP production, mitochondria are also involved in regulating several physiological processes interacting with calcium signaling and reactive oxygen species (ROS) production. Mitochondrial ROS are byproducts of oxidative phosphorylation released primarily due to the Complex I and III activity of the ETC (Murphy, 2009; Chen et al., 2018). ROS is cleared from cells by the action of enzymes like superoxide dismutase (SOD) that modify superoxides to diffusible hydrogen peroxide (H_2_O_2_). Another enzyme, glutathione peroxidase, quenches ROS by oxidizing glutathione. ROS is also scavenged by catalase in peroxisomes (Fransen et al., 2012; Lee et al., 2018). Calcium regulates mitochondrial dehydrogenases such as pyruvate dehydrogenase (PDH), α-ketoglutarate dehydrogenase (α-KGDH), iso-citrate dehydrogenase (ICDH), glyceraldehyde three phosphate dehydrogenase (GAPDH) thereby regulating the ATP production (Brian Glancy, 2012).

The mitochondrial shape is highly dynamic, and they exist in structures ranging from an intricate reticular network to small punctate spheres (Bereiter-Hahn and Vöth, 1994). Their shape is regulated by fission and fusion with the help of dedicated protein machinery of Dynamin-related large GTPases, also aided by cytoskeletal components (Chan, 2006). Mitochondrial fission is carried out by Dynamin-related protein 1 (Drp1), which assembles on the outer membrane and constricts due to GTPase activity, separating the mitochondrion into two daughter mitochondria (Michalska et al., 2018). Mitochondrial fission occurs by initial actin polymerization around the mitochondrion at the fission site followed by Drp1 binding to carry out mitochondrial fission (Korobova et al., 2013). Outer mitochondrial membrane fusion is carried out by Mitofusins 1 and 2 (Mfn1 and 2) known to homo- and hetero-oligomerize (Li et al., 2019). Inner mitochondrial membrane fusion is carried out by Optic atrophy 1 (Opa1). Opa1 also maintains inner membrane cristae morphology (Patten et al., 2014; Quintana-Cabrera et al., 2018). Mitochondrial dynamics and morphology are regulated by controlling the expression levels and activity of fusion and fission proteins (Chang and Blackstone, 2010; Leboucher et al., 2012; Anand et al., 2013).

The mitochondrial shape is regulated in cells based on their energy requirements, cell type and metabolic resources (Kuznetsov and Margreiter, 2009; Lee et al., 2014). Mitochondrial shape and activity are interlinked. Longer mitochondria contain an intricate cristae organization enhancing the ATP output (Paumard et al., 2002; Cogliati et al., 2013). Several studies associate mitochondrial fusion with increased OXPHOS activity (Silva Ramos et al., 2016; Yao et al., 2019), membrane potential (Mitra et al., 2009) and reduction in ROS (Lee et al., 2014). Differentiated cells or tissues such as skeletal muscles (Vendelin et al., 2005; Picard et al., 2013), heart (cardiomyocytes) (Hales and Fuller, 1997; Kasahara et al., 2013; Pennanen et al., 2014; Wai et al., 2015; Kuznetsov et al., 2019), neuronal stem cells (Hales and Fuller, 1997; Ong and Hausenloy, 2010; Pennanen et al., 2014; Wai et al., 2015; Fang et al., 2016) and pancreatic cells (Kuznetsov et al., 2010; Twig et al., 2010) contain reticular mitochondria with increased ATP output. On the other hand, small or fragmented mitochondria, generally found in stem cells and embryonic cells, are relatively poor ATP producers (Motta et al., 2000; Sathananthan and Trounson, 2000; Chen et al., 2012; Westermann, 2012; Lees et al., 2017). Mitochondrial fission is necessary for neuronal differentiation, synapse formation, and embryonic brain development (Ishihara et al., 2009; Wakabayashi et al., 2009) and differentiation of myoblasts (Kim et al., 2013). Growth phase yeast cells, which depend on aerobic respiration, have elaborate mitochondria (Hoffmann and Avers, 1973; Egner et al., 2002). A shift to glycolytic fermentation state resolves the mitochondrial network by fragmentation (Jakobs, 2003). Mitochondria hyperfuse and produce more ATP upon presentation of stress stimuli in the cells (Tondera et al., 2009) and shield them from degeneration (Gomes et al., 2011). Mitochondrial fusion, by inhibition of fission protein Drp1, during starvation stress via mTOR signaling has a similar protective role (Gomes et al., 2011; Rambold et al., 2011). Inhibition of ETC by dissipation of the mitochondrial membrane potential leads to their fragmentation in yeast, mammalian cells, *Drosophila* hemocytes and ovarioles (Legros et al., 2002; Meeusen et al., 2004; Ishihara et al., 2006; Tomer et al., 2018) via Opa1 cleavage and calcium-mediated activation of Drp1 (Cribbs and Strack, 2007; Cereghetti et al., 2008). A decrease in the ATP concentration activates the AMP-Kinase (AMPK) pathway that leads to increased mitochondrial fragmentation followed by cell death (Toyama et al., 2016). The increased cytosolic calcium regulates mitochondrial fragmentation in a ROS-dependent manner suggesting an essential but unresolved role of calcium in regulating the mitochondrial shape in rat cardiomyocytes (Hom et al., 2010).

Mitochondria are transported to distinct cellular locations providing ATP and other metabolites locally. They reach their destinations on the microtubules with the help of motor proteins like Dynein and Kinesin (Pilling et al., 2006). Adaptor proteins such as Miro and Milton aid in the interaction between mitochondria and the motor proteins (Stowers et al., 2002; Górska-Andrzejak et al., 2003; Guo et al., 2005; Glater et al., 2006). Directional mitochondrial transport leading to their polarized distribution has been observed in various tissues such as neurons, muscle cells and *Drosophila* embryos (Baloh et al., 2007; Arribat et al., 2019; Chowdhary et al., 2020). Mitochondrial morphology regulation is vital for their migration. Mitochondrial transport defects have been seen in neurons in mitochondrial fission and fusion mutants, leading to impaired synaptic function (Li et al., 2004; Verstreken et al., 2005). Mitochondrial translocation is abrogated in Drp1 mutant *Drosophila* embryos causing defects in cell formation (Chowdhary et al., 2020). Since mitochondrial morphology regulation is critical for their transport and function, it is likely to play a key role during cell migration and morphogenesis. This review sheds light on the mechanistic analyses pertaining to the role of mitochondria in cytoskeletal regulation during morphogenetic processes ranging from cell formation and division to differentiation to embryogenesis and tissue formation in development and disease across various metazoan species.

## Role of Mitochondrial Morphology and Activity in the Regulation of Epithelial Cell Formation and Epithelial to Mesenchymal Transition

**FIGURE 1:**
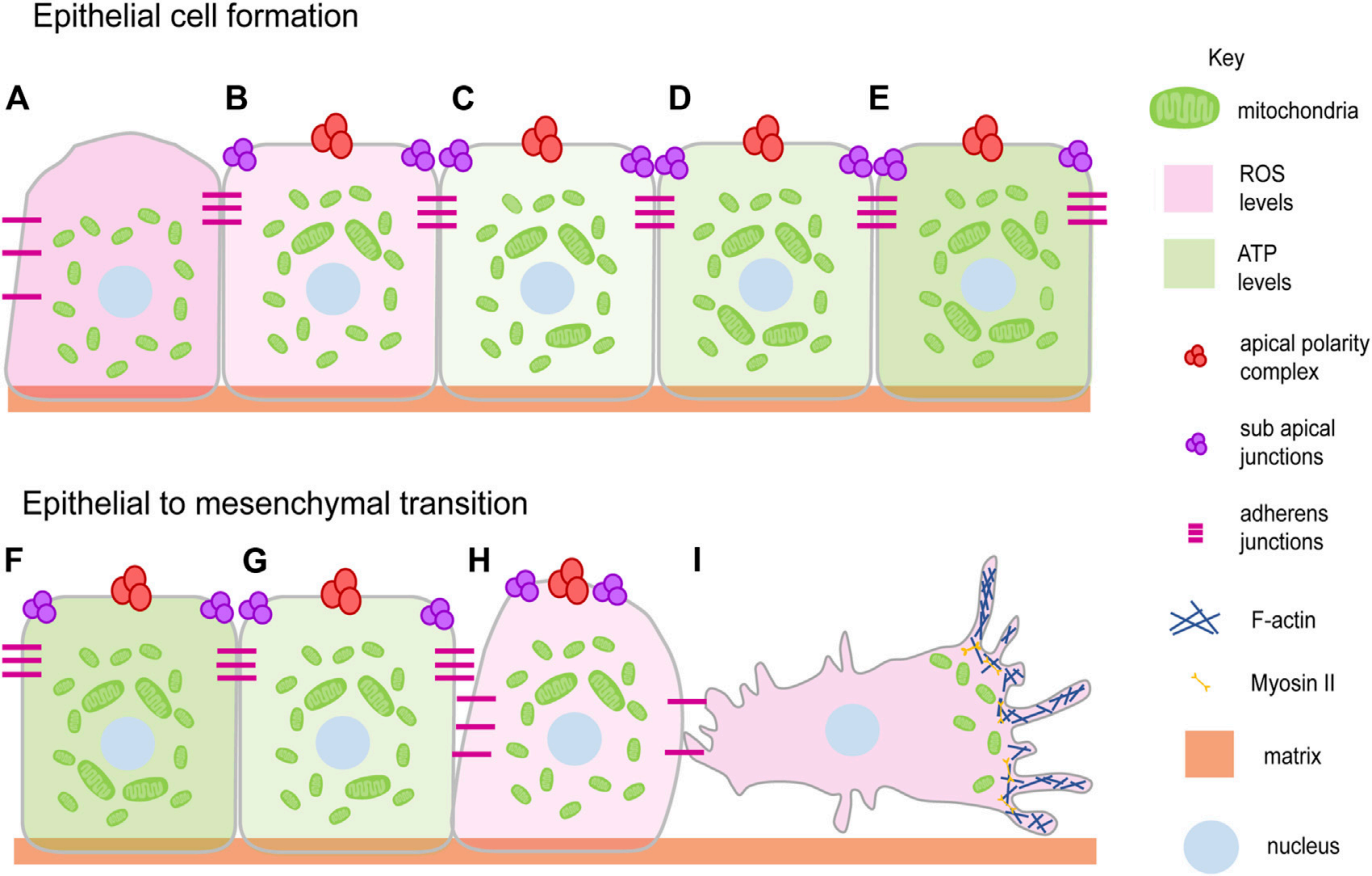
Mitochondrial dynamics and epithelial architecture. Mitochondria transit from spherical to elongated shape during progressive epithelial cell formation and maturation (Fu et al., 2013; Dao Thi et al., 2020) **(A–E)**. This occurs over 6–7 days when liver cell polarization is allowed to occur *in vitro*. Epithelial polarity is lost during epithelial to mesenchymal transition during several processes such as cell migration, wound closure and disease progression with the change in the mitochondrial shape from elongated to spherical **(G–I)**. EMT brings the migratory ability to the cell by detaching and losing polarity complexes (Denisenko et al., 2019; Lee et al., 2019) **(I)**. Mitochondria elongate and show an increase in ATP generating ability (light green gradient) during epithelial cell formation **(B–E)** and become fragmented showing a decrease in ATP generating ability during EMT (Lee et al., 2019) **(F–I)**. The ROS levels decrease (light pink gradient) during epithelial cell formation **(A–E)** and increase during EMT (Zhang et al., 2020; Liu et al., 2020) **(F–I)**. Mitochondria (Green), Apical polarity complexes (Red), Sub apical junctions (Purple), Adherens junctions (Pink), Matrix (Light orange), Nucleus (Light blue).

Epithelial cell formation, differentiation and tissue morphogenesis are regulated by signaling pathways and metabolism during the development of multicellular organisms from a single-celled zygote (Albert Basson, 2012). Epithelial cells form during embryo development or tissue formation by the establishment of cell-cell contacts and recruitment of polarity proteins (Figures 1A–C) (TonyHarris, 2005; Luke Martin McCaffrey, 2012). The process of polarity establishment and maintenance is assisted by cellular processes such as cytoskeletal remodeling, metabolic changes, and the mechanosensory signals associated with them (Vaidžiulytė et al., 2019). The role of mitochondria in the regulation of polarity has been suggested in a variety of tissues (Figures 1A–E). The polarization of hepatic tissue is supported by mitochondrial fusion with increased OXPHOS and ATP production (Figures 1A–E) (Fu et al., 2013). Mitochondria are distinctly localized at conspicuous tight junctions (TJ) in polarized hepatocyte-like cells (HLC) than that of the non-polarized HLC (Dao Thi et al., 2020). Components in TJs are phosphorylated in an ATP-dependent manner in MDCK cells (Tsukamoto and Nigam, 1999). A reduction in the ATP levels in the MDCK cells leads to the formation of large complexes of TJ proteins which associate strongly with the cytoskeleton thereby reducing TJ integrity (Tsukamoto and Nigam, 1997). The CR6-interacting factor 1 (CRIF1) is associated with the mitochondrial OXPHOS and ROS production and is required to regulate TJ assembly via ATP derived from OXPHOS (Ye et al., 1999; Nagar et al., 2014; Lee et al., 2020). Ischemia arising as a result of hypoxia indirectly affects mitochondrial metabolism in the proximal tubule leading to loss of TJ functionality and redistribution of the basolateral membrane lipids and Na-K ATPase (Molitoris et al., 1989). These observations motivate an analysis of how the energy state of a cell affects a specific component of the cell polarity program.

Epithelial to mesenchymal transition (EMT) and cell migration occur on loss of epithelial polarity (Denisenko et al., 2019) (Figures 1F–I). We summarise studies that show how an establishment and a distribution of polarity proteins is dynamically regulated directly or indirectly by mitochondria. Activation of mitochondrial fusion by expression of MFN-1 or inhibition of Drp1 along with PKC-zeta and Numb regulates the asymmetric distribution of mitochondria to regulate stem cell maintenance in the mammary gland cells (Wu et al., 2019). Mutation in Drp1, causing mitochondrial fusion and increased mitochondrial potential, perturbs epithelial cell arrangement in the *Drosophila* follicle cells of the ovaries (Mitra et al., 2012; Tomer et al., 2018). Drp1 also increases ERK2 dependent tumors and in metastatic specimens of breast cancer and lymph nodes of humans (Zhao et al., 2012; Kashatus et al., 2015). The constitutively active p21 and H-RasV12 increase the mitogenic activity with the help of mitochondrial fission and increase in ROS of fibroblast cells based onco-signaling mechanism (Irani et al., 1997; Kashatus et al., 2015). In addition, ROS induces mitochondrial fission and the EMT of the hepatocytes (Zhang et al., 2020). Reduction of mitochondrial ATP also leads to the disintegration of polarity markers such as claudin from the monolayer of the Caco-2 cells (JanssenDuijghuijsen et al., 2017). The increased oxidative stress also leads to the EMT, glycolytic switch in the MCF7 cells (Lee et al., 2019). The tissue-damaging agent, methylenedianiline, increases the biliary epithelial injury and impairs the putative mitochondrial functions prior to losing the tight junction integrity (Santa Cruz et al., 2004). This literature suggests that loss of mitochondrial fission is a mechanism for the maintenance of stemness and increase in mitochondrial fission as a mechanism for promoting mitogenic and metastatic potential (Figures 1F–I).

Besides mitochondrial shape discussed earlier, the mitochondrial metabolites also play an important role in the maintenance of cell shape. Fatty acid oxidation is required for the metastasis of the triple-negative breast cancer cell-TNBC (Camarda et al., 2016). The migration of MDA-MB-435 cells positively depends on aerobic glycolysis (Arseneault et al., 2013). Mitochondrial glutamine metabolism-mediated inhibition of autophagy is essential for the suppression of pancreatic ductal carcinoma while the same metabolism promotes the growth of other cancer cells (Jeong et al., 2016). Lung injury leads to hypoxic conditions that hamper the tissue repair mechanism and cause a reduction in E-cadherin and elevation in EMT markers such as alpha-smooth muscle actin and vimentin in several primary cell lines (Zhou et al., 2009). Further studies on lung cancer cell lines suggest that suppression of mitochondrial function, using drugs such as Oligomycin and Antimycin A, induces mesenchymal markers such as vimentin, snail, and slug and reduces epithelial marker E-cadherin in an AKT-AMPK dependent manner (Han et al., 2018). Metastasis of the different cell lines suggests that cell clustering leading to hypoxia is required to degrade mitochondria and limit the production of ROS to retain survival and metastatic potential (Figure 1I) (Labuschagne et al., 2019). This evidence leaves us with an interesting fact of the involvement of the aerobic and anaerobic metabolism regulating the polarity of the cell and further the shape of the cell and conferring the oncogenic fate. Besides the aerobic or anaerobic mode of regulation, the preference of mitochondria for the metabolites, as reported above for glutamine, creates a curiosity to ask how cancerous transformation happens because of the preferential choice of metabolites.

Mitochondrial calcium buffering activity also plays an important role in various cancers. The hepatocellular carcinoma cell line exhibits the role of mitochondrial calcium uniporter, MCU, in EMT and metastasis through the ROS/Nrf/Notch1 pathway (Jin et al., 2019). The increased growth of colorectal carcinomas is observed as a result of increased uptake of calcium through MCU, increasing mitochondrial biogenesis leading to increased ROS production and it involves ROS/NF-ƙB signaling (Liu et al., 2020). Contrary to that, the loss of calcium uptake by MCU results in loss of polarity and induction of migratory properties in the Hs578t breast cancer cells and HeLa cells (Prudent et al., 2016). There are very few studies on the understanding of the role of ROS and calcium, being secondary messengers, on the polarity of the cell and on the oncogenic transformations.

In summary, the observations cited here show that mitochondrial energy is one of the important factors regulating polarity in epithelial cells or tissues. Apart from that, mitochondrial contribution in the ionic homeostasis such as calcium buffering, and in anabolism by providing important metabolic intermediates help drive the cell fate determination and differentiation process. Having mentioned the regulatory role of mitochondria in the polarity and cell shape aspect here, it is quite fascinating to understand the mechanisms that trigger mitochondria to initiate the process of cell shape change and accomplish them.

## Mitochondrial Morphology and Activity in the Cell Cycle

Eukaryotic cell division involves duplication and segregation of the genetic material as well as of cytoplasm and organelles into daughter cells. The enormous energy requirement for these series of events is primarily fulfilled by glycolysis and OXPHOS. Thus mitochondria play a crucial role in cell division where crosstalk between mitochondria and cell division machinery regulates the different phases of cell cycle.

**FIGURE 2:**
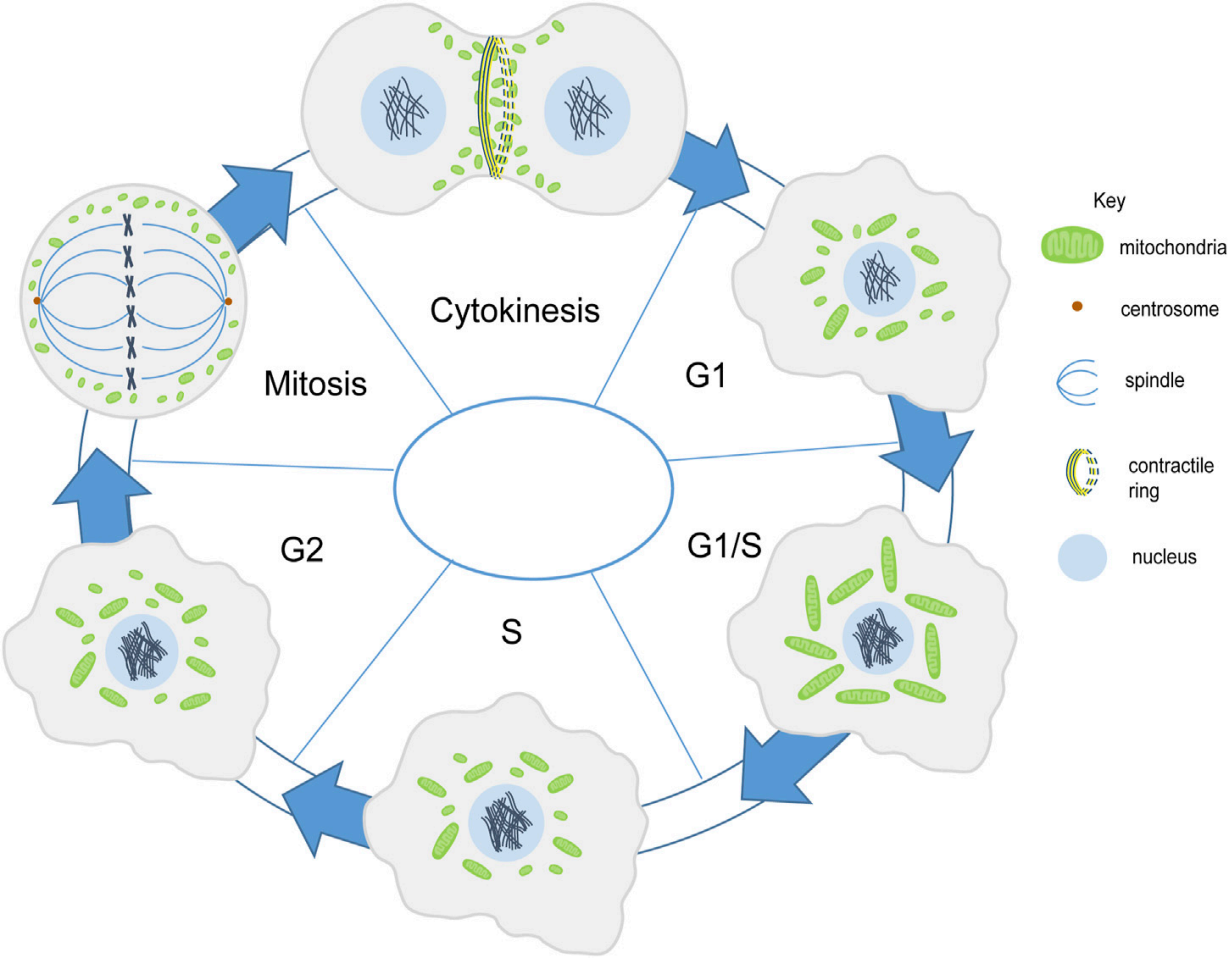
Mitochondria shape change during cell division. Mitochondria (green) are present in fragmented and intermediate morphology during the G1 phase. They form a tubular network in the G1/S phase. In the S phase they become fragmented or have an intermediate morphology (Finkel and Hwang, 2009; Mitra et al., 2009). Mitochondrial membrane potential increases along with mitochondrial elongation from G1 to G2/M phase (Martínez-Diez et al., 2006). Mitochondria get fragmented during mitosis and are further distributed among the daughter cells (Taguchi et al., 2007). Mitochondria align the contractile ring during cytokinesis (Lawrence et al., 2016).

The cell cycle begins with a long growth phase (G1), followed by the S phase where DNA duplication takes place. This is then followed by a shorter growth phase (G2) and finally, the mitotic (M) phase wherein cell division takes place. The regulators of cell cycle include the family of cyclin-dependent kinases (CDKs) and cyclins which together control the progression of the cell from one phase to the next. One of the cell cycle regulators, Cyclin D1, that promotes nuclear DNA synthesis has been shown to regulate mitochondrial size and activity in mice (Wang et al., 2006). Progression through the G1 phase in mammalian cells is accompanied by an increase in mitochondrial membrane potential as measured by TMRE and increase in OXPHOS and ATP production regulated by the mTOR signaling pathway (Schieke et al., 2008). Mitochondria form an interconnected tubular network that is hyperpolarized and shows increase in the oxygen consumption at the G1/S transition (Finkel and Hwang, 2009). This has been suggested to trigger cyclin E induction which is required to proceed to the S phase in mammalian cells (Mitra et al., 2009) (Figure 2). The biosynthesis of cardiolipin, the phospholipid which forms the inner mitochondrial membrane, also takes place in the S phase. The mitochondrial membrane potential has been shown to increase from G1 to the S phase and it increases even further in the G2/M phase (Martínez-Diez et al., 2006). Further, as the cell enters mitosis, mitochondria get fragmented and are distributed among daughter cells. Cdk1/cyclin B complex phosphorylates Drp1 at Ser-585 causing mitochondrial fission in mitosis (Taguchi et al., 2007). Whether this change in mitochondrial morphology and activity is a general feature across all tissues in metazoans remains to be determined.

The distribution of mitochondria during cell division is regulated by the endoplasmic reticulum and cytoskeletal network in various eukaryotic cells such as yeast, several metazoan cells such as scorpions and chironomus insects, mammalian cells and plant cells (Wilson, 1916; Simon et al., 1997; Yaffe et al., 2003; Sheahan et al., 2004; Staiber, 2007; Peraza-Reyes et al., 2010; Rohn et al., 2014). Mitochondria are associated with microtubules during interphase in mammalian Hela cells. However they differentially associate with microtubules in mitosis. Mitochondria dissociate from microtubules in mitosis and this takes place by shedding off of motor proteins, dynein and kinesin mediated by phosphorylation of mitochondrial and cytosolic substrates by CDK1 and Aurora A respectively. Forced binding of mitochondria to motor proteins disrupts mitochondrial distribution and cell cycle progression in mammalian Hela cells (Chung et al., 2016). In later stages mitochondria bind to astral microtubules and accumulate at the cleavage furrow in a microtubule-dependent manner with the help of motor proteins Miro-1 and KIF5B (Lawrence and Mandato, 2013; Lawrence et al., 2016). Further, contractile ring formation and RhoA is essential for mitochondrial localization at the cleavage furrow (Lawrence et al., 2016) (Figure 2); however, the actin cytoskeleton is not required for this tethering (Lawrence and Mandato, 2013). Symmetric segregation of mitochondria in Hela cells is regulated by Myo19 (Rohn et al., 2014). The association of mitochondria with the cytoskeleton is likely to be a conserved mechanism during cell division for equivalent distribution among daughter cells.

Alteration of mitochondrial morphology and function can lead to activation of G1/S cell cycle checkpoints eventually arresting the cells at the G1 phase. Further, mutation in two of the mitochondrial respiratory complexes, Pdsw (complex I) and cytochrome c oxidase (complex IV), reduces cyclin E levels via two independent pathways eventually resulting in G1/S cell cycle arrest in *Drosophila*. The former takes place via increased ROS whereas the latter occurs via decreased ATP production (Mandal et al., 2005, Mandal et al., 2010; Owusu-Ansah et al., 2008). Depolarizing mitochondria, using FCCP treatment, also causes G1/S arrest by triggering p53 dependent cell cycle checkpoint. The transient state of hyperfused mitochondria is essential for increasing cyclin E levels required for S phase entry. However, prolonged hyperfused mitochondrial state causes a delay in entry into the S phase, similar to cyclin E overexpression (Mitra et al., 2009). Hyperfused mitochondrial morphology also causes G2/M arrest and aneuploidy via accumulation of cyclin E (Qian et al., 2012). A part of the total pool of cyclin E is recruited to the mitochondria, which is shown to be regulated by Drp1 and mitochondrial bioenergetics (Parker et al., 2015). Mitochondrial elongation by fission 1 protein (Fis1) depletion also impairs mitotic entry (Lee et al., 2014). These studies indicate that mitochondrial fragmentation is necessary for some mammalian cells to enter mitosis. Depletion of mitochondrially localized syntaphilin increases the number of cells with G2/M DNA content, thereby decreasing the proliferation of tumor cells (Caino et al., 2017).

The role of mitochondrial metabolism in cell division in early embryogenesis has been described in multiple studies with limited systematic analysis of mitochondrial morphology and distribution during different cell cycle steps. Production of mtROS is induced upon fertilization of *Xenopus* embryos. mtROS oscillates with the cell cycle and also keeps the cell cycle phosphatase, Cdc25C, inactive via the redox-sensitive cysteine residues, thereby regulating the cell cycle. Reducing the mtROS using inhibitors such as malonate and sodium azide results in loss of cycling of Cdc25C thereby causing cell cycle arrest during early embryogenesis (Han et al., 2018). *Drosophila* syncytial embryos undergo four rounds of nuclear division accompanied by various morphogenetic movements of the plasma membrane, which require energy from mitochondria (Mazumdar and Mazumdar, 2002; Chowdhary et al., 2017). Mitochondria in these embryos are small and dispersed and distributed asymmetrically along the apico-basal axis with the help of microtubules (Chowdhary et al., 2017). Hypoxia and depletion of OXPHOS by inhibitors and RNAi against ETC components, in *Drosophila* syncytial embryos, reduces ATP levels and results in metaphase arrest (DiGregorio et al., 2001; Kulkarni et al., 2009; Chowdhary et al., 2017). The inhibition of OXPHOS leads to shortening of pseudocleavage furrows in *Drosophila* blastoderm embryos (Chowdhary et al., 2017). Maternal depletion of the epsilon (e)- subunit of the mitochondrial ATP synthase in *Drosophila* embryos causes defects in the cortical divisions. These defects include irregular spacing between nuclei, the abnormal orientation of non-sister centrosomes, and a disorganized actin network reflected by missing metaphase furrows (Kidd et al., 2005). Equal distribution of mitochondria has also been observed in division cycles of the syncytial *Drosophila* blastoderm embryos (Chowdhary et al., 2017).

Alleviation of Drp1 phosphorylation in M phase mammalian cells leads to unequal segregation of mitochondria during division and defects in cytokinesis (Kashatus et al., 2011). Forced fusion of mitochondria using mitochondrial division inhibitor, Mdivi1, the treatment also results in the formation of multinucleate cells (Rohn et al., 2014). These studies suggest that mitochondrial fragmentation is essential for their proper partitioning and for the separation of the two daughter cells undergoing division. Closure of the contractile ring at the base of cells in *Drosophila* cellularization is similar to cytokinesis. The mitochondrial shape has been shown to be important for the ring constriction during cellularization, as Drp1 mutant embryos show expanded rings (Chowdhary et al., 2020). Mdm10, a component of the mitochore complex, upon deletion, results in the formation of multiple buds in budding yeast with mitochondria being present as a large spherical structure in only one of the buds. Also, *mdm10* mutant cells show defects in contractile ring closure, altogether resulting in defects in the separation of the mother and daughter cell (García-Rodríguez et al., 2009).

Multiple studies, *in vivo* and *in vitro*, in different model systems suggest a crucial role of mitochondrial dynamics and activity in the process of cell division. Cell division is accompanied by various shape changes and distinct organization of mitochondria (Figure 2). When cells are rounded during mitosis, microtubules are engaged in spindle formation and mitochondria are fragmented. Defects in cell division in mitochondrial morphology and activity mutants suggest that mitochondria might play a role in driving the cellular and morphological changes in different phases of the cell cycle. Mitochondrial byproducts such as calcium and ROS are important regulators of the cell division machinery (Han et al., 2018). An analysis of whether mitochondrial shape and activity changes occur in all cell types or are a feature of differentiated cells will be compelling to understand the interaction of mitochondria with the cell cycle.

## Cell Migration

**FIGURE 3:**
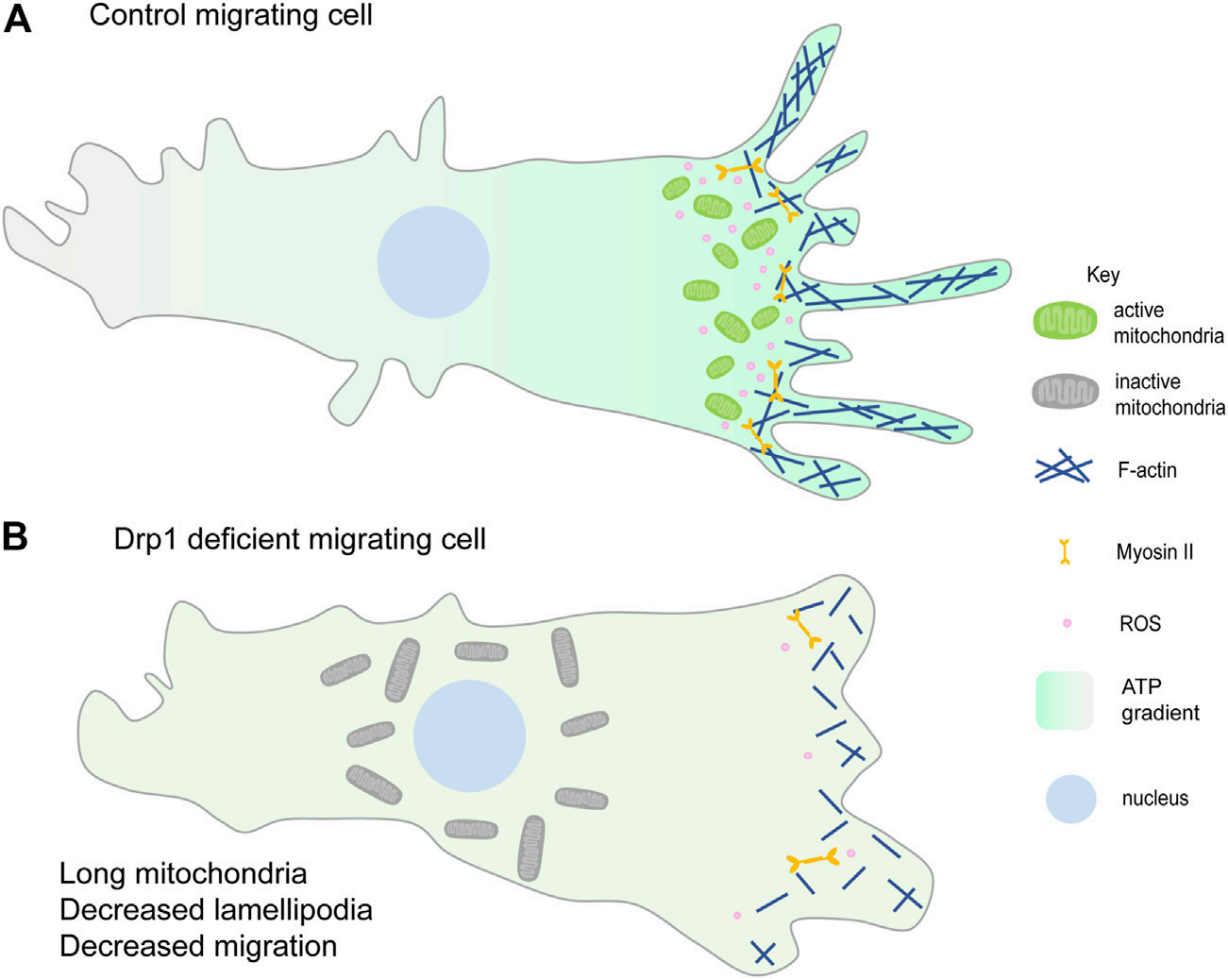
Mitochondrial fragmentation at the leading edge of the migrating cell. **(A)** Fragmented mitochondria (green) migrate towards the leading edge and aid in lamellipodia formation hence promoting migration. Mitochondrial ATP (higher ATP in the migrating cell shown with the green gradient) is essential for lamellipodia formation and hence migration (Zhao et al., 2012; Caino et al., 2016). ROS production (pink) at the leading edge also enhances cell migration (Lee et al., 2018; Ko et al., 2018). **(B)** Loss of mitochondrial fission or increased fusion prevents mitochondrial relocalisation to the leading edge (shown by the perinuclear gray larger mitochondria) and decreases lamellipodia formation thereby decreasing cell migration. Reduced lamellipodia formation could be due to decreased F-actin polymerisation and reduced ROS at the leading edge. Decreasing ROS also decreases cell migration (Wang et al., 2012; Zhao et al., 2012; Desai et al., 2013).

Cell migration occurs during development, immunity, and cancer metastasis and involves dynamic cytoskeletal rearrangements that require mitochondrial energetics and signaling. Migrating cells exhibit various cell shape changes at the migrating front, accompanied by a redistribution of mitochondria at the leading edge. Mitochondria utilize microtubules, motor proteins, Dynein and Kinesin, and adaptor proteins, Miro and Milton, for trafficking within the cell (Fransson et al., 2003; Cox and Spradling, 2006; Pilling et al., 2006). Reorganization of the subcellular localization and structure of mitochondria plays a crucial role in cancer cell metastasis and migration of various cell types such as lymphocytes, neutrophils, smooth muscle cells, neural stem cells (Zhao et al., 2012; Sun et al., 2018). Improper spatial distribution of mitochondria, seen in Miro-1 downregulated mouse embryonic fibroblasts, results in migration defects and lowered stability of focal adhesion (Desai et al., 2013; Schuler et al., 2017). The metastatic and migratory potential of ovarian cancer cells is also reported to be regulated by the AMPK based mitochondrial distribution at the leading edge (Cunniff et al., 2016). Lowering of syntaphilin (SNPH), a neuronal cytoskeletal protein that regulates mitochondrial movement causes relocalization of mitochondria from perinuclear position to the cortical cytoskeleton and leads to increased tumor cell motility (Caino et al., 2016; Caino et al., 2017; Seo et al., 2018) (Figure 3A). In summary, differential distribution of mitochondria at specific locations imparts migratory potential to the cell.

Mitochondrial fragmentation is essential for their efficient translocation towards active zones in migrating cells. PDGF-induced vascular smooth muscle cell (VSMC) migration and lamellipodia formation require Drp1 mediated mitochondrial fission (Lim et al., 2015; Wang et al., 2015). Mitochondrial fission facilitates chemokine-induced lymphocyte migration (Campello et al., 2006). Consistent with this scenario, cancer cell metastasis has been associated with increased mitochondrial fission in several independent studies. Loss of mitochondrial fragmentation or increased fusion suppresses breast cancer cell and epithelial cancer cell migration (Zhao et al., 2012; Desai et al., 2013) (Figure 3B). Increased mitochondrial fission, by silencing of Mfn proteins, increases lamellipodia formation and recruitment of mitochondria to lamellipodia regions in breast cancer cells (Zhao et al., 2012). dAKAP1, protein kinase A-anchoring protein, sequesters protein kinase A (PKA) onto the outer mitochondrial membrane, inhibits Drp1 by phosphorylation and leads to mitochondrial fusion. Breast cancer cells with low dAKAP1 expression are more motile and have mitochondria with low membrane potential. Inhibiting Drp1 activity in adult neural stem cells (aNSC) alters the migratory elongated cell morphology to an irregular cuboidal shape, thereby disrupting their migration and differentiation (Kim et al., 2015).

In contrast, some studies suggest an opposing relationship between cell migration and mitochondrial fission. Secreted frizzled-related protein 2 (SFRP2) inhibits non-small lung cancer cell (NSCLC) proliferation and metastasis via activating mitochondrial fission (Li et al., 2019). MFN2 regulates neutrophil migration *in vivo* in zebrafish and mice. Knocking down Mfn2 in human neutrophil-like cells results in a reduction of mitochondria-ER tethers which leads to an increase in cytoplasmic calcium and Rac hyperactivation eventually decreasing the neutrophil migration (Zhou et al., 2020). *Mfn*2 null MEFs are spherical with membrane ruffles in contrast to elongated *wt* MEFs which have transient filopodia and lamellipodia while spreading (Zhou et al., 2020). MFN2 is shown to have a non-canonical role in negatively regulating tumor cell invasion. Knockdown of MFN2 promotes phosphorylation of mTORC2 which increases phosphorylation of Akt finally leading to increased metastasis (Xu et al., 2017).

The coordinated and dynamic movement of mitochondria along the migration front(s) ensures spatio-temporal regulation of ATP production, ROS levels and calcium buffering required for cellular migration. Disruption of mitochondrial ATP synthesis, using drug treatment, reduces F-actin polymerization and lamellipodia formation in breast cancer cells thereby suppressing their migration abilities (Zhao et al., 2012) (Figure 3A). However, loss of mitochondrial function using inhibitors of mitochondrial respiration promotes cell migration via increased cytosolic calcium and intracellular ROS levels (Chang et al., 2009; Hung et al., 2012). Inhibition of mitochondrial ETC using drugs, such as oligomycin A or antimycin A, changes the morphology of epithelial lung cancer cells to mesenchymal-like spindle shape, eventually enhancing their migration and invasion abilities as well. It was shown that induction of EMT by mitochondrial respiratory inhibitors was mediated by activation of AKT and AMPK pathways (Han et al., 2018). Mitochondrial superoxide dismutase (SOD2) upon overexpression scavenges superoxide radicals and decreases Ang II-induced VSMC migration via a decrease in Akt phosphorylation (Wang et al., 2012) (Figure 3). Production of ROS is seen from complex I and III of mitochondrial ETC, in liver cells, upon stimulation with TNF-a. mtROS further increases the cell migration of the liver cells via activation of the NF-kB signalling (Kastl et al., 2014). The reduced level of mitochondrial deacetylase, SIRT3, and increased ROS level in the leading edge of breast epithelial cells leads to activation of Src and focal adhesion kinase (FAK) signaling to promote collective cell migration (Lee et al., 2018). The invasive property of cancer cells is enhanced in hydrogen peroxide-inducible clone-5 (HIC-5) knockdown by increased stability of MMP9 mRNA via NADPH oxidase 4 (NOX4) mediated mtROS (Mori et al., 2019). Depletion of mitochondrial calcium uniporter (MCU) decreases cell migration in various breast cancer cells (Tang et al., 2015; Prudent et al., 2016; Tosatto et al., 2016). Knockdown of MCU causes defective cell migration during zebrafish gastrulation via deregulation of actin dynamics (Prudent et al., 2013). Succinate, a citric acid cycle metabolite, promotes human mesenchymal stem cell migration via regulating mitochondrial morphology and activity. Succinate causes Drp1 phosphorylation that leads to mitochondrial fission and increases mtROS levels which further leads to F-actin formation finally leading to increased migration (Ko et al., 2018). Nuclear respiratory factor 1 (Nrf1), mitochondrial biogenesis regulator, tissue-specific knockout mice show defective neurite outgrowth and retinal ganglion cell migration during retinal development (Kiyama et al., 2018). Various components of the Hippo pathway have been shown to regulate migration via altering mitochondrial activity. Mst1 upon overexpression increases ROCK1 levels which decreases F-actin expression and increases mitochondrial apoptosis, mtROS levels finally leading to decreased migration (Zhang et al., 2018). However, YAP, another component of the Hippo pathway, is shown to have a positive correlation with human rectal cancer cell migration (Li et al., 2017).

Cell migration is one of the many processes which derives energy from mitochondrial metabolism. Mitochondrial morphology has also been shown to be critical for cell migration in various different tissues. Mitochondria are redistributed to the migrating front of the cells to provide energy or metabolites for cytoskeletal remodeling that aids in cell shape changes that accompany their movement.

## Wound Healing

**FIGURE 4:**
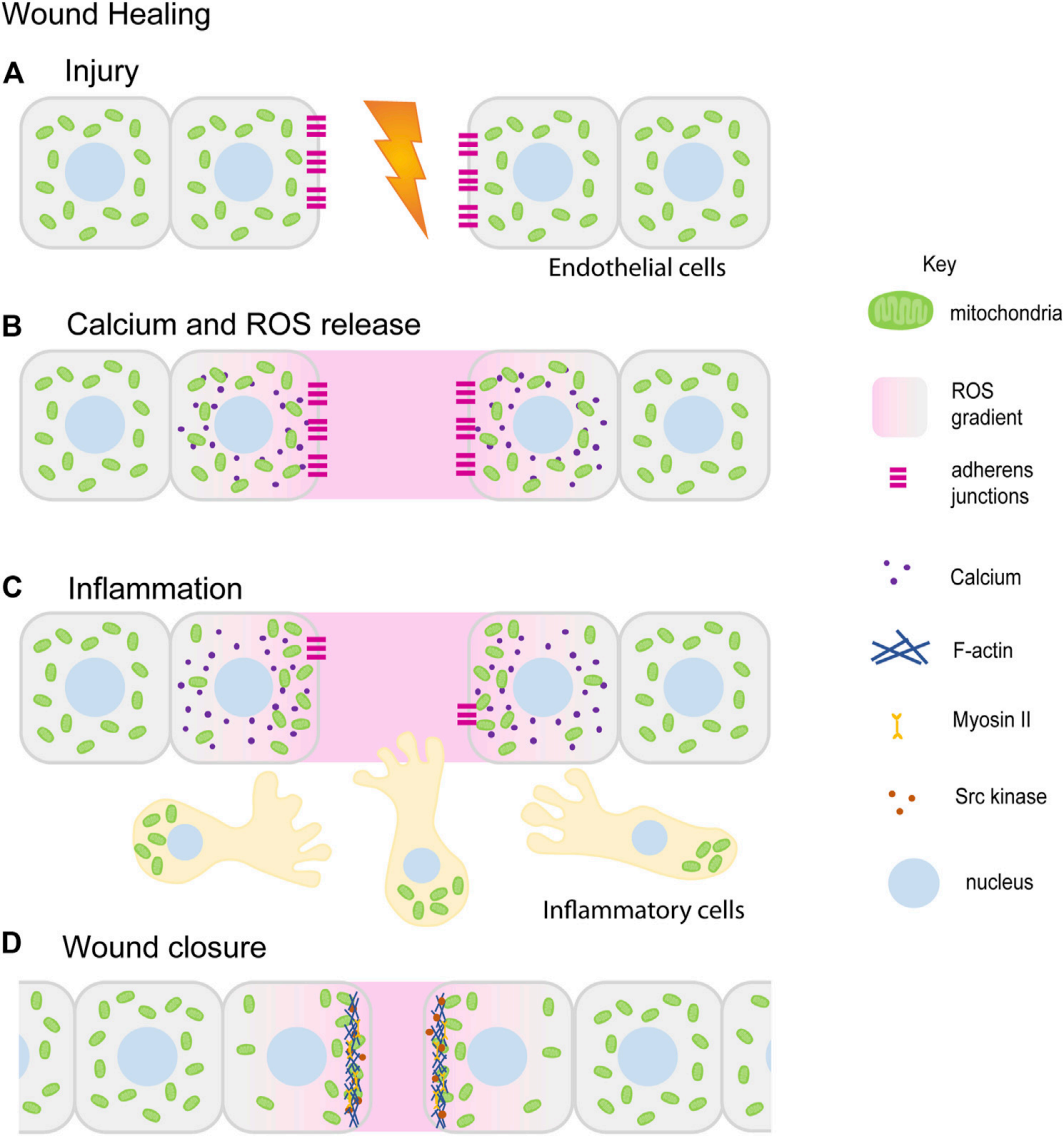
Mitochondrial redistribution and function in wound healing. The schematic represents an overview of mitochondrial contribution in the series of events during the wound healing process. Upon injury, cells in the damaged tissue lose contact with each other forming a lesion in the tissue **(A)**. In response to the wounding, cytoplasmic calcium (purple) and ROS (pink) levels increase locally at the wound site ROS (pink) is also released extracellularly at the wound site **(B) **(Wright et al., 2008; Guo et al., 2017; Guo et al., 2020). Small, fragmented mitochondria (green) in cells adjacent to the wound site redistribute towards the wound site. Inflammatory cells and immune cells (beige) migrate towards the wound site in response to ROS. Mitochondria (green) are localized at the uropods of these cells **(C)** (Campello et al., 2006; Morlino et al., 2014). This is followed by tissue morphogenesis and closure aided by increased actomyosin (blue–Actin, yellow–Myosin) based constriction and loss of E-cadherin (magenta) **(C,D)** (Fu et al., 2020; Ponte et al., 2020). Only a few of the references that have led to the figure compilation have been cited here.

Healing and repair of wounds involves a coordinated role for multiple cell types, chemical and mechanical cues, cytoskeleton and morphogenesis. It is a multi-step process that mainly involves: hemostasis, inflammation, and tissue remodeling. Hemostasis is the first step in wound healing that is essential for the closure of blood vessels to stop bleeding and coagulation. Wounding of the epithelial tissue initiates rapid remodeling and proliferation of inner lining of blood vessels consisting of endothelial cells (ECs) and undergo neoangiogenesis during injury under the influence of angiogenic factors such as Vascular Endothelial Growth Factor (VEGF) and angiopoietin (Kim et al., 2000; Gerhardt et al., 2003; Hellström et al., 2007; De Bock et al., 2013). The leading cells or the tip cells among these ECs respond to the growth factor cues, remodel their cytoskeleton and migrate in the direction of the tissue injury eventually leading to the blood vessel branching (Ruhrberg et al., 2002; Cascone et al., 2003; Hellström et al., 2007; Bodnar, 2015). Simultaneously, inflammatory responses are recruited at the wound site to protect the injured tissue from pathogens. In response to chemoattractant signals released at the wound site, inflammatory cells such as neutrophils and macrophages migrate towards the wound (Redd et al., 2006; He and Marneros, 2013; Su and Richmond, 2015). Finally, the cells in the wounded tissue proliferate and re-epithelialize to seal the wound (Jameson et al., 2004; Gurtner et al., 2008; Hunter et al., 2018). Mitochondrial shape and signaling aids cell migration, proliferation and tissue constriction by modulating cellular cytoskeleton during these wound healing steps as described further below (Figure 4).

### Hemostasis

Angiogenic factors such as VEGF that are required for blood vessel remodeling or neoangiogenesis are released at the wound site in response to hypoxic conditions and mtROS (Forsythe et al., 1996; Chua et al., 1998; Sen et al., 2002; Kastl et al., 2014; Harel et al., 2017). Mitochondrially localized novel protein transduction domain of Ubiquinol–cytochrome c reductase binding protein (PTD-UQCRB) increases ROS that induces VEGF expression *in vitro* and speeds up angiogenesis and wound healing in mice (Chang et al., 2015). Angiogenic factors such as VEGF and angiopoietin also stimulate mitochondrial biogenesis (Wright et al., 2008), OXPHOS activity (Guo et al., 2017; Diebold et al., 2019) and mtROS production at the wound sites (Wang et al., 2011; Harel et al., 2017) (Figures 4A,B).

Earlier studies showed that ECs contain nascent mitochondria and their metabolism is largely dependent on glycolysis (Krützfeldt et al., 1990; Culic et al., 1997; De Bock et al., 2013; Cruys et al., 2016). However, a number of relatively recent studies show that the role of mitochondrial ETC and OXPHOS is essential. Glycolysis in the EC tip cells is essential for their differentiation whereas OXPHOS in non-tip ECs plays a role in cell survival, transcriptional activities and ROS production (Yetkin-Arik et al., 2019). Mitochondria in the ECs become elongated upon proangiogenic stimuli by the upregulation of Mfn (Lugus et al., 2011; Zhou et al., 2015; Ren et al., 2020) and Opa1 activity (Xu et al., 2019; Herkenne et al., 2020) and downregulation of Drp1 and Fis1 (Xu et al., 2019; Ren et al., 2020). Opa1 also regulates the activity of NF-ƙB and is essential for the proliferation and migration of ECs *in vitro* (Herkenne et al., 2020). Inhibition of mitochondrial ETC complex III reduces proliferation of ECs *in vitro* and blocks postnatal retinal angiogenesis in mice (Diebold et al., 2019). EC specific knockout of *cox10* (heme-O-farnesyl transferase, COX, Complex IV) causes embryonic lethality in mice. Cox10 KO adult mice show defects in vascularization near wounds (Schiffmann et al., 2020). Inhibition of mitochondrial biogenesis also leads to impaired vasculature in mouse kidney injury models. Promoting the biogenesis by activating 5-hydroxytryptamine (5-HT, serotonin) 1F receptor, a G-protein coupled receptor, leads to increased migration and branching in human and mouse ECs *in vitro* (Dupre et al., 2019).

In addition to inducing the expression of angiogenic factors, ROS reportedly regulates cytoskeletal activity in ECs to facilitate their migration towards wound sites. ROS induces actin polymerization at cell margins in cultured endothelial cells (Moldovan et al., 2000; Ikeda et al., 2005; Kaplan et al., 2011). Actin stress fibers are evident in the case of bovine pulmonary artery endothelial cells upon ROS exposure (Vepa et al., 1999). An increase in Myosin light chain kinase phosphorylation is also observed in ECs upon ROS induction (Zhao and Davis, 1998; Usatyuk et al., 2012). VEGF-induced increase in ROS levels in mouse endothelial cells leads to increased Rac1 activity (Deshpande et al., 2000; Wang et al., 2011). Rac1 is a Rho-GTP binding protein involved in the regulation of actin polymerization and lamellipodia formation at the leading edges of migrating cells (Katoh et al., 2006; Ridley, 2006). On the other hand, quenching ROS by overexpression of catalase decreases phosphorylation of Rac1 and downstream signaling components p21-activated kinase (PAK), AKT, p38 MAPK, and ERK (Wang et al., 2011) which are essential for proliferation and migration of ECs towards the wound. VEGF can regulate the production of ROS through JNK/ERK and PKC pathways by modulating the levels of adaptor protein p66Shc, a stress response protein involved in longevity. p66Shc regulates Rac1 activity downstream of ROS (Oshikawa et al., 2012). ROS also influences Rac1 mediated disassembly of vascular-endothelial (VE)-cadherin and induces migration by reducing cell-cell adhesion (van Wetering et al., 2002). Mechanical stimuli can also increase ROS production in ECs (Wung et al., 1997; Ali et al., 2006). Focal Adhesion Kinases (FAKs) are essential for integrin binding and actin stress fiber formation in migrating cells (Rousseau et al., 1997). mtROS induces phosphorylation of FAKs through PKC signaling in cultured human ECs (Abedi and Zachary, 1997; Gozin et al., 1998), bovine pulmonary artery ECs (Vepa et al., 1999) and in mechanically disrupted EC cells (Ali et al., 2006). Additionally, mtROS can amplify PKC signaling itself directly (Lee et al., 2004; DelCarlo and Loeser, 2006). ROS also leads to phosphorylation of paxillin in cultured ECs (Abedi and Zachary, 1997; Gozin et al., 1998). Paxillin is the adapter that binds the focal adhesions to the actin cytoskeleton in the cells (Turner, 2000).

### Inflammation

Several factors including increased ROS at the wound site serve as key chemoattractants for recruitment of immune response cells towards the wound. mtROS also aids in the production of pro-inflammatory signaling molecules such as cytokines, regulation of TNF-α signalling (Wang et al., 2009; Bulua et al., 2011; Rowlands et al., 2011), activation of NFkB (Chandel et al., 2000) and inflammasomes (Zhou et al., 2010; Zhou et al., 2011) (Figure 4C). On the other hand, mtROS production at wounds is also induced by inflammation through TNF-α signalling (Klyubin et al., 1996; Corda et al., 2001; Yang et al., 2007; Kastl et al., 2014). Extracellular ROS is necessary for recruitment of leukocytes in zebrafish tailfin injury (Niethammer et al., 2009). H_2_O_2_ and ROS serve as chemoattractants for hemocytes at wounds in *Drosophila* embryos (Moreira et al., 2010; Razzell et al., 2013). Mitochondria reorganize at the leading front infection synapse of natural killer (NK) cells. Mitochondria depolarize upon presentation of cancerous cells or infection signals (Abarca-Rojano et al., 2009). Localization of small but active mitochondria at NK cell synapses is dependent on Drp1 (Quintana et al., 2007; Baixauli et al., 2011). Mitochondrial migration towards the uropods is mediated by Miro-1 and Dynein motors on microtubules in lymphocytes (Campello et al., 2006; Morlino et al., 2014). Inhibition of mitochondrial transport impairs chemoattractant directed lymphocyte migration (Morlino et al., 2014). Drp1 silencing reduces p-MLC levels and thereby actomyosin activity at immune synapses of T-cell lymphoblasts (Baixauli et al., 2011).

### Tissue Remodelling

The role of ROS in cell proliferation and wound healing has been largely discussed in cell lines as well as animal model systems. Burst of mtROS is observed locally at wound sites in *Caenorhabditis elegans*, *Drosophila* and zebrafish epidermal cells (Niethammer et al., 2009; Xu and Chisholm, 2014; Hunter et al., 2018; Fu et al., 2020), *Xenopus* embryos (Han et al., 2018), adult skin (Roy et al., 2006) as well as in cell culture systems (Ross et al., 2011). ROS is also upregulated during dorsal closure during *Drosophila* embryogenesis. This process zippers the embryonic epithelial sheet at the dorsal midline and involves cellular reorganization similar to wound healing (Muliyil and Narasimha, 2014). Small and fragmented mitochondria are targeted towards the wound site in cells undergoing wound closure (Croft and Tarin, 1970; Muliyil and Narasimha, 2014; Hunter et al., 2018; Crakes et al., 2019). On the contrary, elongated mitochondria are present in shoulder tendon injuries. Changes in the mitochondrial architecture are accompanied by increased mitochondria activity and biogenesis (Thankam et al., 2018; Crakes et al., 2019). Enhanced cytoplasmic calcium (Ca^2+^) levels are essential for Drp1 dependent mitochondrial fragmentation at the wound site (Ponte et al., 2020). ROS is also shown to be responsible for mitochondrial fragmentation (Muliyil and Narasimha, 2014). Drp1 independent mitochondrial fragmentation occurs via Fzo1 depletion with the help of a Miro1 and is essential for the process of wound healing (Fu et al., 2020). Cell migration and wound healing speeds are significantly slowed down in Miro1 mutant mouse fibroblasts in scratch assays (Schuler et al., 2017). Additionally inhibiting mitochondrial fragmentation leads to a reduction in Ca^2+^ and ROS at the wound sites and delays the process of healing (Razzell et al., 2013; Muliyil and Narasimha, 2014; Ponte et al., 2020).

Rapid tissue remodeling during wound healing demands high levels of O_2_ and surplus mitochondrial ATP production and is also an indicator of mechanical damage in the tissues (Caporossi and Manetti, 1992; Pastor et al., 1992). This ATP production and release is essential for wound closure (Handly and Wollman, 2017). ATP release occurs by increasing intracellular ATP delivery speeds, the immune response, cytokine production and re-epithelialization (Mo et al., 2019). This is also accompanied by increased levels of collagen that facilitate faster wound healing (Sarojini et al., 2017; Mo et al., 2019). Transfer of healthy mitochondria to bone marrow transplant increases cell proliferation and migration to improve bone defect healing (Guo et al., 2020). The release of ATP is likely to be through mechanosensory ion channels such as TRPV4 (Mihara et al., 2011; Boudaka et al., 2020). Deletion of TRPV4 in esophageal keratinocytes enhances wound healing further (Boudaka et al., 2020) by increasing cell migration rates. ATP production stimulates Ca^2+^ release upon membrane rupture in wounds (Romanello et al., 2001; Robling and Turner, 2009; Lopez-Ayon et al., 2014; Handly and Wollman, 2017; Mikolajewicz et al., 2018) in PKC dependent manner (Mikolajewicz et al., 2018). Calcium signaling through the Mitochondrial Permeability Transition Pore regulates PTEN signaling and is essential for wound closure (Marcu et al., 2015).

Cellular re-organization and re-epithelialization at the wound site is driven by pulsed actomyosin contractile activity (Muliyil and Narasimha, 2014; Razzell et al., 2014; Hunter et al., 2015). Removal of E-cadherin from the wound margin is essential for actomyosin assembly at the wound site (Carvalho et al., 2014; Hunter et al., 2015; Matsubayashi et al., 2015). Inhibition of ROS at the wound sites increases E-cadherin levels preventing junctional remodeling (Hunter et al., 2018). ROS can chemically alter protein activity by oxidizing disulfide linkages (Yang et al., 2007). Phosphatases and kinases containing cysteines are key targets for ROS-induced modification (Brandes et al., 2009; Fedorova et al., 2010; Steinberg, 2013). ROS regulates localization of E-cadherin, oxidation of zebrafish Scr Kinase Lyn (Yoo et al., 2012) and in *Drosophila*, orthologous kinase Src42A (Takahashi et al., 2005). ROS and Ca^2+^ signaling at the wounds is required for F-actin localization at the wound (Figure 4D). Drp1 mutant embryos containing reduced levels of ROS and mitochondrial Ca^2+^ have defective F-actin assembly and considerably delayed wound closure (Ponte et al., 2020). F-actin regulation is likely via Ca^2+^ dependent activation of RhoGTPase effector Protein kinase N (Pkn) (Mukai et al., 1997; Ponte et al., 2020). ROS production also enhances the activity of actin and Myosin II in the epidermal cell wounds and during dorsal closure by activating upstream kinases such as Rho1, ROCK and Src. Rho1 gets oxidized at cysteine residues by ROS and inhibited (Xu and Chisholm, 2014) to promote Myosin II independent ring closure in wound healing in syncytial cells in *C. elegans*. Src kinase on the other hand (Hunter et al., 2018) gets activated on oxidation by ROS for Myosin II dependent wound closure in epithelial cells in *Drosophila* (Figure 4D).

Multiple observations have given insights into the likely regulation of mitochondrial morphology, localization and activity during the multiple steps of the wound healing program. Mitochondria fragment and accumulate at high activity demanding regions in migrating angiogenic and inflammatory cells, and in constricting cells for healing the tissue. ROS, ATP production and calcium are key mitochondrial signals that regulate cytoskeleton and tissue morphogenesis in wounds. Studies with these preliminary candidates will in future elucidate mechanisms of mitochondrial control of wound healing signalling in embryos and complex tissues in future.

## Embryonic Development and Tissue Formation

### Mitochondrial Selection and Morphology Dynamics During Early Embryogenesis

Mitochondria are maternally inherited in most metazoan embryos. Mitochondria along with other organelles aggregate to form a cloud-like structure or fusome or Balbiani body in oocytes of metazoans such as mouse (Pepling et al., 2007), human (Hertig, 1968), beetle (Jaglarz et al., 2003), *Xenopus* (Chang et al., 2004), zebrafish (Kaufman et al., 2018), chick (Ukeshima and Fujimoto, 1991) and *Drosophila* (Cox and Spradling, 2003; Cox and Spradling, 2006) and are inherited in the embryos.

Healthy mitochondria with relatively higher membrane potential selectively aggregate in Balbiani bodies of *Xenopus* (Wilding et al., 2001a), zebrafish (Zhang et al., 2008), *Drosophila* (Hill et al., 2014; Lieber et al., 2019), mouse (Van Blerkom et al., 2003; Dalton and Carroll, 2013) and human (Wilding et al., 2001b; Van Blerkom, 2011) oocytes. This selection works in the favor of biogenesis of healthy mitochondria to attain a critical mtDNA copy number (Callen et al., 1980; Reynier et al., 2001; Wai et al., 2010; Hurd et al., 2016; Lieber et al., 2019) and against those containing severe mtDNA mutations (Fan et al., 2008; Stewart et al., 2008; Hill et al., 2014). The transport of healthy mitochondria depends on oskar in *Drosophila* oocytes (Hurd et al., 2016; Lieber et al., 2019). However, mitochondria, in general, are nascent and contain decreased levels of ETC complexes with low ATP activity in *Drosophila*, amphibian, fish, mouse and human oocytes (Wallace and Selman, 1990; Trimarchi et al., 2000; Dumollard et al., 2007; Pepling et al., 2007; Van Blerkom, 2011; Sieber et al., 2016). Mature *Drosophila* and *Xenopus* oocytes depend on glycolysis for their energy demands and accumulate glycogen under the influence of glycogen synthase kinase 3 (GSK3) signaling and reduced insulin (Sieber et al., 2016). During fertilization, mitochondrial OXPHOS activity and Kreb’s cycle is triggered upon sperm entry that induces Ca^2+^ waves in starfish, ascidian and mouse eggs (Roegiers et al., 1995; Schomer and Epel, 1998; Dumollard et al., 2003; Dumollard et al., 2004; Campbell and Swann, 2006) and is essential for embryonic competence and survival (Van Blerkom et al., 1995; Wilding et al., 2001a; Ottosen et al., 2007).

During fertilization, sperm brings very few mitochondria into the oocytes and these are subject to degradation either during spermatogenesis or post-fertilization. mtDNA of paternal mitochondria is degraded before fertilization with the help of mitochondrial endonuclease G in *C. elegans* (Zhou et al., 2016) and *Drosophila* (DeLuca and O’Farrell, 2012) and mediated by mitochondrial DNA polymerase in *Drosophila* (Yu et al., 2017). In paternal mitochondria that carry mtDNA into the oocytes, mtDNA ubiquitination guides their removal by autophagy and lysosomal degradation after fertilization during early stages of embryogenesis as observed in *Drosophila* (Politi et al., 2014), *C. elegans* (Zhou et al., 2011; Sato and Sato, 2011; Merlet et al., 2019), mouse (Al Rawi et al., 2011; Song et al., 2016) and pigs (Sutovsky, 2003; Sutovsky et al., 2003). Mitochondrial fission and loss of membrane potential have been recently shown to play a role in marking them for degradation in *C. elegans* embryos (Wang et al., 2016). These mechanisms ensure the uniparental inheritance of mtDNA.

Embryogenesis, followed by fertilization, proceeds through various developmental stages such as the formation of a blastula and gastrulation which represent varying mitochondrial morphology. Mammalian blastocyst stage embryos have small and punctate mitochondria with perinuclear distribution as observed across multiple model systems such as human (Motta et al., 2000; Sathananthan and Trounson, 2000; Van Blerkom et al., 2000; Wilding et al., 2001b; Acton et al., 2004), hamster (Squirrell et al., 1999), primate (Squirrell et al., 2003), porcine (Katayama et al., 2007). These mitochondria contain relatively less defined cristae (Akiyama and Okada, 1992; Sathananthan and Trounson, 2000; Romek et al., 2011). During human embryogenesis, with increasing cell numbers and cellular activity, mitochondria appear elongated (Sathananthan and Trounson, 2000) with elaborate cristae architecture (Stern et al., 1971; Sathananthan and Trounson, 2000). A rapid increase in mitochondrial biogenesis, mtDNA number, OXPHOS activity is also observed in the placentation stage in rat embryos (Alcolea et al., 2007a; Alcolea et al., 2007b). Mitochondrial biogenesis and OXPHOS activity show an increase during zebrafish embryogenesis (Stackley et al., 2011). Although most studies clearly indicate the presence and need for mitochondrial energy, a few reports also suggest that ATP through mitochondria is minimal in human and mouse early embryos (Bavister and Squirrell, 2000; Motta et al., 2000) and mitochondrial bioenergetic enzymes are found in the nucleus where they regulate transcriptional activation (Nagaraj et al., 2017). Thus the above observations of mitochondrial morphology and the associated metabolic changes motivate further investigation on how mitochondria regulate different stages of embryonic development and regulation of transitions from one stage to another stage along with metabolic changes.

Similar to mammalian embryos, mitochondria are small and perinuclear in early *Drosophila* embryos and their activity is uniform across the syncytial cells from anterior to posterior during early and late blastoderm stages. A detailed analysis of metabolite levels shows an increase in TCA cycle intermediates in the *Drosophila* embryos (Tennessen et al., 2014). 0–4 h embryos largely seem to utilize amino acids such as Glutamate and Aspartate that are metabolized through ETC (An et al., 2014). Pharmacological and genetic inhibition of ETC activity shows energy stress as indicated by elevated pAMPK fluorescence in *Drosophila* embryos (Chowdhary et al., 2017). These reports together indicate that despite the small structure, mitochondria in blastoderm stage *Drosophila* embryos are metabolically active. The above observations show that smaller and fragmented mitochondria are also active.

The balanced activity of mitochondrial fission and fusion proteins is essential for embryonic development and survival. Loss of mitochondrial fusion proteins Mfn1/2 and Opa1 in mouse embryos causes lethality (Chen et al., 2003; Zhao et al., 2015). Mfn2 mutant embryos also contain placental defects (Chen et al., 2003). Opa1 mutant mouse embryos with fragmented mitochondria also exhibit retarded growth and early embryonic lethality (Moore et al., 2010). Drp1 mutant mouse embryos are also embryonic lethal and display defects in neural tube formation and defective brain development (Ishihara et al., 2009; Wakabayashi et al., 2009). Embryonic lethality has also been observed in Drp1 and Opa1 knockdown *Drosophila* embryos (Chowdhary et al., 2020). Mfn2 knockdown substantially reduces mitochondrial membrane potential and ATP levels thereby inducing Bcl2 dependent apoptosis in mouse blastoderm embryos due to decreased Ca^2+^ levels (Zhao et al., 2015). Somatic cell nuclear transfer (SCNT) embryos usually have lower success rates of transfer and development. Controversially, Mfn1 overexpression increases the developmental capacity of bovine SCNT embryos by improving ATP synthesis rates and reducing hydrogen peroxide production despite having a mitochondrial shape different from other embryos (Hua et al., 2012).

### Asymmetric Mitochondrial Activity Requirement During Embryogenesis

Non-uniform or asymmetrically distributed mitochondrial activity has been reported in a variety of embryo model systems. Mouse and human preimplantation embryos consist of mitochondria with high membrane potential located in their subcortical regions as reported using mitochondrial potential dye JC1 (Wilding et al., 2001b; Van Blerkom et al., 2002; Van Blerkom et al., 2003; Nagaraj et al., 2017). Other studies using mitotracker or TMRE dye do not report such a distinction in mitochondrial activity (Nishi et al., 2003; Dumollard et al., 2004; Newhall et al., 2006). Dye-dependent discrepancies in mitochondrial membrane potential readout may be observed due to the differential impact and sensitivity of the dyes to tissues (Perry et al., 2011). Differences in mitochondrial activity across embryonic axes have been reported in a variety of model systems. Sand dollar embryos, exposed to asymmetric gradients of respiratory inhibitors, polarize into the oral-aboral axis based on the redox gradient (Pease, 1941). The cytoplasmic streaming due to sperm triggered Ca^2+^ waves at fertilization in *Xenopus* and ascidian oocytes (Savage and Danilchik, 1993; Roegiers et al., 1995; Dumollard et al., 2006), establishes an asymmetry of mitochondrial distribution and function across animal-vegetal poles. (Roegiers et al., 1995). Similarly, the oral axis in sea urchin embryos has higher mitochondrial density and therefore has more redox activity compared to the aboral axis (Coffman et al., 2004; Coffman et al., 2009). The redox activity differences and hypoxia-inducing factor α (HIFα) may also regulate Nodal signaling in sea urchin embryos by controlling transcription activity on the dorsal side of the embryos (Coffman et al., 2014; Chang et al., 2017). Aggregation of mtlr-RNA, indicative of mitochondrial density, was observed at the prospective dorsal marginal zone in *Xenopus* embryos (Yost et al., 1995). A dorso-ventral gradient of mitochondrial membrane potential was observed in *Drosophila* embryos using a fluorescent dye JC1 (Schiffmann, 1997). Posterior pole plasm in *Drosophila* embryos has a stronger accumulation of Rhodamine 123 indicating higher activity of mitochondria (Akiyama and Okada, 1992). These studies together suggest a developmental signaling coupled regulation of mitochondrial distribution and activity.

**FIGURE 5:**
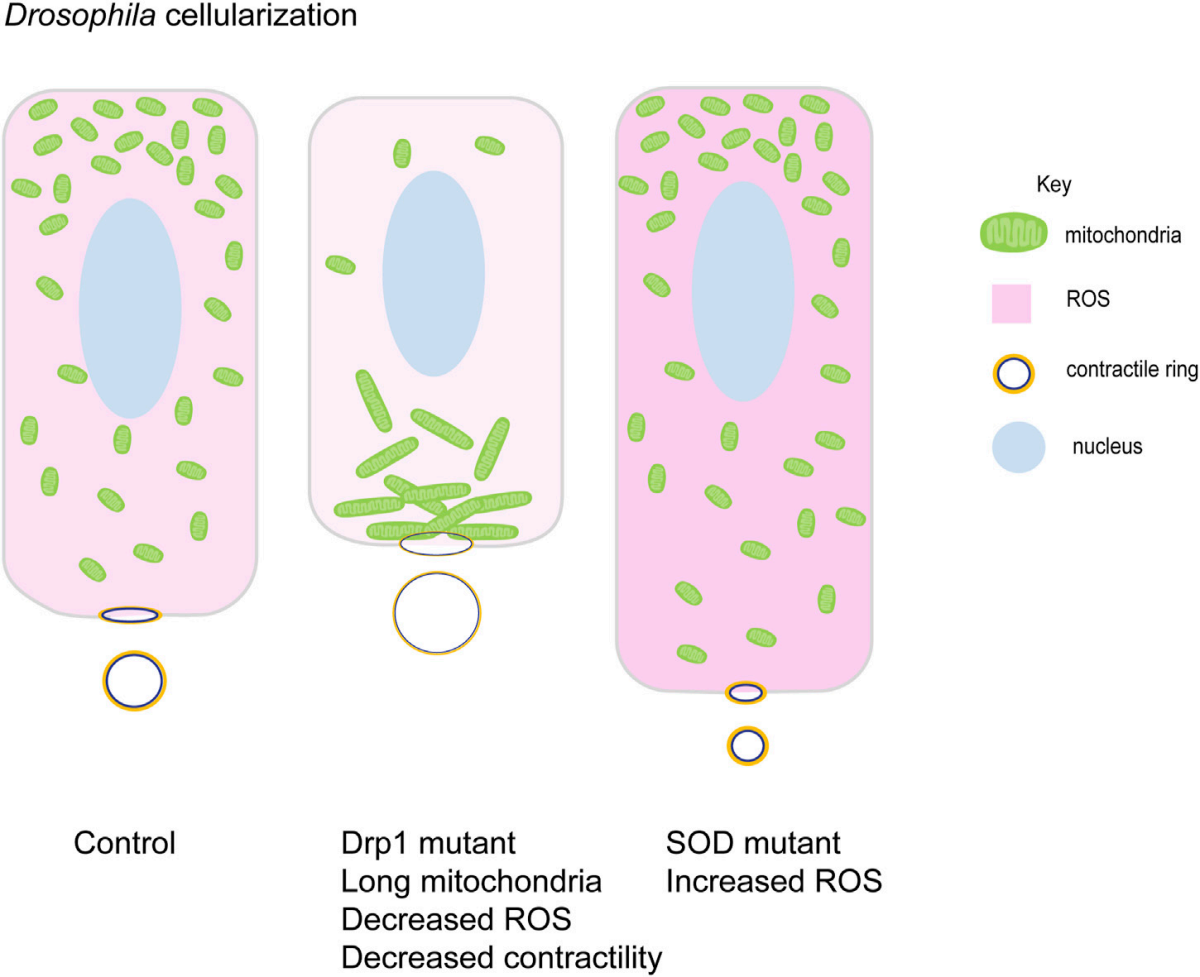
Mitochondrial morphology function in cellularization in *Drosophila* embryogenesis. Small round mitochondria migrate apically during cellularization in *Drosophila* embryos. Schematic represents the terminal timepoints of cellularization in control, Drp1 mutant and SOD mutant embryos. Light to dark pink shades represent increasing levels of ROS in these cells. Control embryos have apically migrated small mitochondria (green). Myosin II (yellow) is localized at basal contractile rings. Clustered mitochondria (green) in Drp1 mutant embryos accumulate basally and fail to migrate apically. Lowered ROS levels (light pink), lead to decreased Myosin II (yellow) and contractility at the contractile rings. These cells are shorter than controls with large contractile rings. SOD mutant embryos have high ROS levels (dark pink) and small mitochondria (green) are present apically. These cells show increased Myosin II (yellow) levels with increased contractility and smaller rings. They are taller compared to controls (Chowdhary et al., 2020).

The asymmetric mitochondrial localization and activity indicate their specialized functions in specific regions and timepoints of rapid morphogenetic changes during embryogenesis. However, a precise role for heightened mitochondrial localization or activity is yet to be explored in detail. Actin structures and cell architecture are disorganized in cryopreserved and vitrified sheep embryos with reduced mitochondrial activity (Dalcin et al., 2013). Mitochondrial ATP is required for maintaining spindle orientation (Kidd et al., 2005), metaphase furrow formation (Chowdhary et al., 2017) in nuclear division cycles of *Drosophila* syncytial blastoderm embryos. Inhibition of ATP synthesis in zebrafish embryos arrests the process of gastrulation (Pinho et al., 2013). Mouse embryo knockouts of mitochondrial ribosomal proteins involved in ATP production have abnormal mitochondrial morphology, show delayed development and fail to gastrulate (Cheong et al., 2020). Active mitochondria are present in the yolk syncytial layer of zebrafish blastula stage embryos. An apoptotic regulator Nrz, localized to mitochondria, mediates actomyosin contractility via Ca^2+^ during zebrafish epiboly (Popgeorgiev et al., 2011). Another proapoptotic protein, Bcl-wav localizes to mitochondria and regulates mitochondrial calcium uniporter (MCU). Knockdown of Bcl-wav reduces actin protrusions and retards cell movements in zebrafish epiboly (Prudent et al., 2013). Ca^2+^ waves induced during fertilization also induce mtROS production via MCU in *Xenopus* embryos. mtROS regulates cell cycle progression by activating Cdc25C phosphatase in blastoderm embryos and inhibition of mtROS delays the cell cycle (Han et al., 2018). The oral side of sea urchin embryos consists of more mitochondrial density, leading to more ROS production in this region. Reduction of ROS by injecting ROS quenching enzyme catalase reduces expression axis specific TF nodal by reducing p38 oxidation and abrogates development (Coffman et al., 2009). ROS levels also show an increase during zebrafish gastrulation. Reduction of ROS levels by inhibition of NADPH oxidase (Nox) leads to E-cadherin mislocalization which blocks cell movements during zebrafish epiboly (Mendieta-Serrano et al., 2019). ROS is essential for balanced actomyosin contractile activity during *Drosophila* cellularization. Drp1 mutant embryos, having reduced ROS levels, contain cells with reduced sqh (myosin light chain) levels and less contractile actomyosin rings (Figure 5). Enhancing ROS activity in these embryos speeds up cellularization, forming taller cells with highly constricted rings at their basal regions (Chowdhary et al., 2020). Similarly, balanced mtROS levels are essential for the regulation of Myosin II activity in cell constriction and delamination during dorsal closure in *Drosophila* embryos (Muliyil and Narasimha, 2014). As indicated from the literature, mitochondrial activity and distribution are regulated during morphogenetic processes during development and are likely to strongly contribute to the regulation of cytoskeletal remodeling in early embryos. This necessitates further investigation to analyze a precise link between mitochondria and developmental signaling.

Embryogenesis shows organogenesis to create organized tissues. The reports in different animal models have shown the inclusive role of mitochondria in tissue and organ development to some extent such as somite formation, neuronal differentiation and heart development. In zebrafish embryos mitochondria have a homogeneous distribution in the early somitogenesis stage (18 hpf), in later stages, they redistribute to the somite boundaries and become elongated (24 hpf). The mitochondrial distribution becomes homogeneous again by 36 hpf (Arribat et al., 2019). Mitochondrial migration towards somite boundaries is dependent on Miro. Dnm1 mutants lack mitochondrial trafficking. Electron transport chain supercomplexes accumulate and increase 18 hfp onward. This indicates a gradual increase in mitochondrial activity. Inhibition of ATP production delays embryo development (Arribat et al., 2019). Nrz, one of the proteins associated with mitochondria, plays a role in the cellular movement during somitogenesis in the zebrafish (Arnaud et al., 2005). The Yin yang protein required for the mitochondrial gene expression is an important regulator for the villi development in the intestinal epithelium of mouse and OXPHOS genes are also required for the villi maturation and intestinal development (Kumar et al., 2016).

## Conclusion and Future Perspectives

Mitochondrial shape and activity are integral to morphogenetic processes such as cell migration, cell division, cell differentiation, wound healing and disease, especially cancer. Changes in ROS, calcium, ATP, mitochondrial metabolites and mitochondrial shape are key determinants for driving cell shape and identity changes during the various processes discussed in the review. ROS affects signaling pathways by oxidizing key signaling molecules. The oxidation changes the propensity of these signaling components to undergo regulatory post-translational modifications such as phosphorylation. It will be interesting to use various biochemical methods to identify a cohort of such proteins whose activity could be modulated by oxidation by varying ROS levels in cells. Some actin remodeling proteins, in addition to the actomyosin complex, have been shown to be modified by ROS. Analysis of other cytoskeletal regulators likely to be oxidized by ROS needs to be carried out. Metabolic products from the TCA cycle and fatty acid oxidation are also likely to be involved with cell shape changes. Mitochondrial shape and activity changes during cell division have been well studied, and their perturbation causes cell cycle arrest. However, whether cell shape changes and cytokinesis during cell division are affected by mitochondrial shape and activity remains to be investigated. It would be essential to study and report the mitochondrial effects on cytoskeletal components during cell division using dynamic live imaging reporters. Several studies discuss the importance of mitochondrial shape, ATP and ROS during cell migration. Collectively, they suggest that small mitochondria migrate towards the cellular leading edge to provide ATP, ROS and other metabolites for assisting the migration process and the cell shape changes accompanying it. However, a detailed mechanistic analysis of mitochondrial regulation and its targets is needed. More *in vivo* studies in model systems and non-model systems will provide a comprehensive analysis of some of the universal mechanisms of interaction of mitochondrial morphology and activity involved in morphogenetic changes during differentiation and development. Usage of *in vivo* live imaging markers in model systems for visualizing mitochondrial dynamics and improved metabolic sensors for glucose, pyruvate, alpha-ketoglutarate, citrate and lactate as well ATP and ROS would serve as some of the key ways to understanding the dynamicity of mitochondrial morphology, localization and function during development (Plaçais et al., 2017; Zhang et al., 2020; Keller et al., 2021) also enabling better design for diagnostics and therapeutic interventions in development and disease.
